# Insomnia and anxiety among COVID-19 patients in China: the chain mediating effect of psychological capital and self-esteem

**DOI:** 10.1186/s12912-023-01563-8

**Published:** 2024-04-01

**Authors:** Juan Du, Chao Wu, Wen-Kai Zheng, Sheng-Nan Cui, Ling Li, Zhuo Liu, Li Gao, Chun-Ni Heng, Hong-Juan Lang

**Affiliations:** 1 School of Nursing, the Fourth Military Medical University, Xi’an, 710032 China; 2 School of Basic Medicine, Inner Mongolia Medical University, Hohhot, 010110 China; 3 Department of endocrinology, Tangdu Hospital, Fourth Military Medical University, Xian, Shaanxi 710038 China

**Keywords:** Insomnia, Psychological capital, Self-esteem, Anxiety, Chain mediating effect, COVID-19

## Abstract

**Background:**

The outbreak of Corona Virus Disease (COVID-19) in 2019 has continued until now, posing a huge threat to the public’s physical and mental health, resulting in different degrees of mental health problems. As a vulnerable segment of the public, anxiety is one of the most common mental health problems among COVID-19 patients. Excessive anxiety aggravates the physical and psychological symptoms of COVID-19 patients, which is detrimental to their treatment and recovery, increases financial expenditure, affects family relations, and adds to the medical burden.

**Objective:**

This study aimed to explore the role of psychological capital and self-esteem in the relationship between insomnia and anxiety, thereby shedding light on the mechanism of the effect of insomnia on anxiety in COVID-19 patients.

**Methods:**

A cross-sectional study was conducted from April to May 2022 in Fangcang hospital in Shanghai, China. The self-administered questionnaires were distributed to 718 COVID-19 patients via cell phone using the Internet platform “Questionnaire Star”, which included Athens Insomnia Scale, Psychological Capital Questionnaire, Self-esteem Scale, Self-Rating Anxiety Scale, gender, age, marital status, education. Data analysis was performed using descriptive analysis, independent-samples t-test, one-way analysis of variance, Pearson correlation analysis, ordinary least-squares regression, and bootstrap method.

**Results:**

Education background had significant impact on anxiety in COVID-19 patients (F = 7.70, *P* < 0.001). Insomnia, psychological capital, self-esteem and anxiety were significantly correlated, respectively (*P* < 0.001). And Regression analysis showed that insomnia had a direct negative predictive effect on psychological capital (β = -0.70, *P* < 0.001) and self-esteem (β = -0.13, *P* < 0.001). Psychological capital had a direct positive predictive effect on self-esteem (β = 0.12, *P* < 0.001). Insomnia had a direct positive predictive effect on anxiety (β = 0.61, *P* < 0.001). Both psychological capital and self-esteem had significant negative predictive effects on anxiety (β = -0.06, *P* < 0.05; β = -0.72, *P* < 0.001). The results showed that the mediating effect of psychological capital and self-esteem was significant, and the mediating effect value was 0.21. First, the indirect effect consisting of insomnia - psychological capital - anxiety was 0.04, showing that psychological capital had a significant mediating effect. Second, the indirect effect consisting of insomnia-self-esteem-anxiety had a value of 0.10, indicating that self-esteem had a significant mediating effect. Third, the indirect effect consisting of insomnia-psychological capital-self-esteem-anxiety had a value of 0.06, suggesting that psychological capital and self-esteem had a significant chain mediating effect between insomnia and anxiety.

**Conclusions:**

Insomnia had a significant positive predictive effect on anxiety. Insomnia was first associated with a decrease in psychological capital, followed by a sequential decrease in self-esteem, which in turn was associated with increased anxiety symptoms in COVID-19 patients. Therefore, focusing on improving the psychological capital and self-esteem of patients can help alleviate the anxiety caused by insomnia in COVID-19 patients. It is recommended that patients and health care professionals increase the psychological capital and Self-esteem of COVID-19 patients through various methods to counter the effects of insomnia on anxiety.

## Introduction

The outbreak of Corona Virus Disease (COVID-19) in 2019 has been continuing till now, posing a great threat to the public’ physical and mental health, resulting in different degrees of mental health problems, such as anxiety, depression, insomnia, traumatic stress disorder and suicidal ideation [2]. The long-term effects of COVID-19 have exacerbated public mental health problems. As a vulnerable group in the public, COVID-19 patients have many psychological fluctuations, such as fear, worry and anxiety, due to changes in their original life patterns and the influence of disease factors during hospitalization, and even cause varying degrees of emotional distress and mental pain, mainly anxiety and fear [48]. Studies have found that the risk of anxiety in the epidemic period is significantly higher than that in the non-epidemic period [35]. The global pandemic of COVID-19 has led to global hunger. Recessions and declines, conflicts and instability have a negative impact on global security. Specifically, the epidemic has disrupted the stability and smoothness of the global food supply chain, weakened the ability of vulnerable countries and populations to access food, and exacerbated political instability and conflict. Especially due to various factors such as age and culture, some older people may be less likely to accept something new and still maintain old ideas, which leads to their relative lag in the cognition and use of digital technology [49].The anxiety and panic caused by information lag affect the sense of crisis of the elderly as high-risk groups and digital vulnerable groups, as well as the burden brought by diseases to the body. Multiple factors lead to patients as vulnerable groups in the outbreak of COVID-19.Previous studies have shown that the fear of disease and the pathological state of the body could aggravate the anxiety symptoms of COVID-19 patients, and the poor sleep state of COVID-19 patients during hospitalization was also one of the important reasons for their anxiety [6, 22, 30]. Although the relationship between insomnia and anxiety in COVID-19 patients has been empirically demonstrated during the COVID-19 pandemic [16], there has been little research on the mechanisms by which insomnia causes anxiety. The aim of this study is therefore to explore the mechanisms underlying the relationship between insomnia and anxiety, thereby providing new ideas for the design of pathways to improve patient anxiety.

### Insomnia and anxiety

Anxiety is the most common mental health problem in the world, which refers to a kind of emotional experience produced by individuals’ cognitive evaluation of their inability to cope with internal and external stimuli that threaten their self-esteem. The causes of anxiety are mainly related to physical pressure, mental challenges or both [47]. For COVID-19 patients, centralized isolation and treatment are required. In addition to the stress and anxiety associated with the disease, anxiety may also be aggravated by centralized isolation and treatment, dense population, unfamiliar environment, cohabitation of COVID-19 patients, depressing environment of the ward, family members and loved ones suffering from COVID-19 or worsening of the disease or death due to COVID-19 [2, 40]. According to relevant data, the incidence of anxiety in COVID-19 patients was about 30.8%–46.2% [9]. Mild anxiety is an adaptive emotional response, which has been shown to have protective effects and is helpful for healthy development, but excessive anxiety can have adverse effects. For individuals, this affects their social role, productivity at work, physical health and mental health; For families, this increases financial costs and affects family relationships; for society, studies have found that over-anxious people have significantly higher demand for primary health care (including physical examination, expert consultation, and frequency of emergency and ambulance calls) than non-anxious patients, resulting in uneven distribution of primary health care [9, 40, 42, 47]. Epidemiological studies have shown that about 50% of people with anxiety disorders were affected by sleep disorders, especially insomnia, which could be triggered or further exacerbated by sleep deprivation [5]. Insomnia is a kind of sleep disorder characterized by frequent difficulty in falling asleep or difficulty in maintaining sleep, resulting in insufficient sleep satisfaction. Prolonged-term insomnia will not only reduce the quality of life and affect social function, but also cause a series of physical and mental problems, such as cardiovascular and cerebrovascular diseases, metabolic diseases, tumors, and anxiety [9]. Furbish et al. found that insomnia significantly predicted anxiety [9, 18, 19]. Studies found that insomnia was negatively correlated with anxiety [44]. Meng et al. found that anxiety was closely related to poor sleep in COVID-19 patients [29]. Because the sleep quality could not be guaranteed, inpatients were frequently in a state of stress, prone to emotional fluctuations, and prone to anxiety when encountering difficulties [4]. Insomnia could be an independent predictor of anxiety [28, 33]. These empirical studies clearly show that insomnia can have a negative effect on anxiety.

However, the mechanisms behind this relationship between insomnia and anxiety in the presence of a pandemic remain largely unexplored. People with COVID-19 may experience additional severe sleep disturbances during the pandemic, increasing the risk of insomnia affecting anxiety. Previous studies on the influencing factors of anxiety mostly focused on chronic stress, occupational stress, fear, life frustration, neurological disorder and other adverse factors [10], and generally ignored the potential impact of individual positive psychological qualities on anxiety. Luthans emphasizes that people’s positive psychological strength is an internal psychological resource that can be developed and improved. It emphasizes the development and management of people’s psychological advantages so as to promote individual understanding and explore their own potential [26]. Therefore, it is necessary to explore the underlying factors in the relationship between insomnia and anxiety in COVID-19 patients from the perspective of positive psychology to find new ideas for anxiety interventions.

### Psychological capital and self-esteem as potential mediating factors

Psychological capital is defined as“a positive mental state of an individual”, which contains four core components: self-efficacy, hope, optimism and resilience [25] The adjusted invasion hypothesis provides an influential theoretical basis for studying the association between insomnia and anxiety through psychological capital [19]. According to this theory, initial externalizing problems, such as insomnia, affect future susceptibility to internalizing symptoms, like psychological capital, which in turn aggravate anxiety problems [19]. Simultaneously, studies have shown that insomnia would reduce individual psychological capital, and was negatively correlated with psychological capital [8]. As an individual positive psychological resource, psychological capital played the role of “buffer” in the formation of negative emotions such as anxiety and depression and could resist individual anxiety and depression symptoms [15]. Low psychological capital could easily induce anxiety and other negative emotions [15, 17]. Thus, it has been conjectured that psychological capital may act as a mediator between insomnia and anxiety. Self-esteem refers to an individual’s perception, judgment and acceptance of his or her own ability and value as a whole [1]. Self-esteem is closely related to one’s emotions, behavior and cognition. Individuals with high self-esteem have strong social adaptation ability, which helps individuals to adjust their mental state and reduce the problem of somatization tendency in the face of difficulties [31]. Low self-esteem will weaken the ability of individuals to adapt to society, and when faced with difficulties, individuals are prone to negative psychological emotions (such as anxiety), which will affect their mental health [11]. Sun et al. found that insomnia was inversely correlated with self-esteem, and insomnia was positively correlated with anxiety in patients with colorectal cancer [44]. Hasani et al. found that self-esteem was negatively related to anxiety [11], and Lin et al. also found that insomnia during the epidemic would significantly affect anxiety level [23]. Therefore, it is speculated that self-esteem may play a mediating role between insomnia and anxiety.

In terms of the impact on self-esteem, from the perspective of positive psychology, certain psychological qualities help people in distress to live a more satisfactory and dignified life, and effectively reduce or even eliminate the trouble caused by adverse factors [22]. The psychological capital of COVID-19 sufferers is just such a positive psychological resource. It refers to the psychological ability that COVID-19 patients possess to promote social adaptation, which is composed of optimism, hope, resilience, self-efficacy and other factors, and covers the basic connotation of psychological capital elements [32]. According to the Conservation of Resources (COR), individuals will become relatively vulnerable when resources are insufficient, thus experiencing additional psychological pressure and negative emotions; when resources are sufficient, they have better coping ability and a stronger sense of self-worth and ability [14]. Studies have found that psychological capital and all its dimensions have positive predictive effects on self-esteem. This suggests that the higher the level of psychological capital, the stronger the sense of self-esteem. Combined with the above speculation, psychological capital and self-esteem may have a chain mediating effect between insomnia and anxiety.

### Purpose of this study

​Based on the above analysis, this study intends to explore the relationship between insomnia, psychological capital, self-esteem, and anxiety in COVID-19 patients, thereby shedding light on the mechanism of the effect of insomnia on anxiety in COVID-19 patients. Based on existing relevant studies, this study puts forward the following hypotheses: (1) Insomnia would be positively correlated with anxiety in COVID-19 patients; (2) Psychological capital would play a mediating role between insomnia and anxiety; (3) Self-esteem would play a mediating role between insomnia and anxiety; (4) Insomnia in COVID-19 patients would have an impact on anxiety through the sequential mediating effect of psychological capital and self-esteem.

## Materials and methods

The cross-sectional survey was conducted from April to May 2022 in Shanghai, China. Convenience sampling was used to recruit participants with confirmed COVID-19 diagnosis at Fangcang Hospital who were informed of the purpose of the study. And questionnaires via cell phone using the Internet platform “Questionnaire Star” were sent out after obtaining informed consent. Inclusion criteria were those between 18 and 65 years who were (1) able to understand and complete the questionnaire independently; (2) voluntarily and with informed consent; (3) no mental disorder. Exclusion criteria: (1) patients with organic brain lesions; (2) patients who did not have basic reading and comprehension skills. The questionnaire was filled out anonymously.

### Sampling and sample size

Firstly, the sample size required for the study was calculated by using the cross-sectional survey sample size estimation formula n = t^2^_α_*P*Q ÷ d^2^, where n represents the sample size, α represents the level of significance which is usually taken as 0.05 or 0.01 (0.05 was taken for the present study), t is the value of the statistical measure which is about 1.96 (fixed value when α is taken as 0.05), P is the expected presenting prevalence rate, Q = 1-P, and d is the tolerable error. Based on the prevalence of anxiety in COVID-19 patients as 31.79% [39], with the tolerance error d taken as 4%, the sample size required for the study was calculated to be about 521 cases, considering the possibility of 10% invalid responses, it was determined that at least 579 respondents were required for the survey. Actually, a total of 718 questionnaires were collected, and 31 were removed (5 with less than 300 seconds completed, 1 apparently wrong, 10 with high homogeneity, and 15 outside the age range). Finally, 687 effective questionnaires were used.

### Data collection

The data collection process for this study employed an online method. The Internet platform “Questionnaire Star” were used for researchers to sent out questionnaire to eligible COVID -19 patients after obtaining informed consent. Before questionnaire, the researchers provided detailed explanations to the participating patients regarding the purpose of the study, the expected outcomes, and the importance of their involvement. It was clearly highlighted that participation in this study was entirely voluntary and participants had the option to withdraw at any point without facing any negative consequences. In addition, the confidentiality and data protection measures of the study are clearly stated, ensuring full respect for participants’ privacy.

### Measures

#### Basic information

The self-developed demographic questionnaire included gender, education, age, and marital status.

#### Measurement of Insomnia

The AIS developed by Hamiltony in 1959 was applied in this study [43]. This questionnaire included 8 items and each item was rated from 0 (none) to 3 (severe), and the score range for insomnia was 0–24, with higher scores indicating poorer sleep quality. It is an internationally recognized self-rating scale of sleep quality. Studies have showed that this scale had excellent reliability and validity [30]. The Cronbach’s alpha coefficient of the scale was 0.884 in this study.

#### Measurement of Psychological Capital

Psychological Capital Questionnaire developed by Luthans et al. in 2007 was used to measure the participants’ psychological capital [26]. There were four dimensions (6 items, respectively): self-efficacy, hope, resilience, and optimism, including 24 items total, with each item scored on a 6-point Likert scale, from 1 (extremely unsatisfactory) to 6 (extremely satisfied). The range for psychological capital score was from 24 to 144, with a higher total score for the subject indicating a higher psychological capital level. Previous studies have shown that this scale was in excellent reliability and validity [54]. The Cronbach alpha coefficient for the scale in this study was 0.942.

#### Measurement of self-esteem

The SES developed by Rosenberg in 1965 was utilized to measure the overall feelings of self-worth and self-acceptance in this study [38]. There were 10 items on this scale and each item was graded on a 4-point Likert scale, from 1 (firmly disagree) to 4 (firmly agree), and item 3, 5, 9, and 10 were reverse scored. The self-esteem level was expressed as a total score ranging from 10 to 40, with higher scores indicating higher self-esteem. In previous studies, this scale showed excellent reliability and validity [53]. The Cronbach alpha coefficient for the SES in this study was 0.756.

#### Measurement of anxiety

SAS developed by Zung et al. in 1971 was utilized to measure participants’ anxiety [57]. This questionnaire included 20 items and each item was graded on a 4-point Likert scale, from 1 (never) to 4 (always). The total score was calculated by the sum of the scores for each item, and the standard score of this scale was utilized to measure anxiety levels (standard score = total score * 1.25 and rounded to the nearest whole number). Previous studies have shown that the scale had excellent reliability and validity [45]. The Cronbach alpha coefficient for this scale in this study was 0.853.

### Statistical methods

Gaussian distribution of data was tested using Kolmogorov-Smirnov (K-S) single sample test and P-P plot. The descriptive analysis of general demographic data was expressed as count (n) and percentage (%); the effect of general demographic data on anxiety was analyzed by independent samples *t* test and one-way analysis of variance (ANOVA). Pearson correlation analysis was performed on four variables (insomnia, psychological capital, self-esteem, and anxiety). The mediating role of psychological capital and self-esteem in the relationship between insomnia and anxiety was examined using the PROCESS macro model 6 established by Hayes [12], with all demographic data as covariates in the analysis. The method is based on ordinary least-squares regression and bootstrapping. Hayes suggested the use of 10,000 bootstrap bias-corrected 95% confidence intervals (BC CIs) for mediation analysis in tests from serial-multiple mediation Model 6, which were considered significant if they did not contain zero [12]. The unstandardized path coefficients were computed to reduce type-1 errors due to distribution. SPSS 25.0 software was used for statistical analysis, and the level of significance was *P* < 0.05.

## Results

### Univariate analysis of general demographic information on anxiety

Descriptive analysis, independent sample t-test and one-way analysis of variance were used to describe and compare the general demographic data (gender, age, marital status, education) and the distribution of anxiety scores, as shown in Table 1. The results of one-way ANOVA showed that education background of COVID-19 patients (F = 7.70, *P* < 0.001) had significant influence on anxiety, as shown in Table 1.

**Table 1:** Effects of general demographic data on anxiety

Category		n	Percentage	SAS	*t/F*	*P*
Gender	Male	418	60.8%	44.26 ± 8.98	0.483	0.167
	Female	269	39.2%	45.23 ± 8.97		
Age(years)	≤ 20	52	7.6%	45.02 ± 9.07	0.225	0.925
	20–30	177	17.0%	44.52 ± 8.00		
	31–40	202	29.4%	44.29 ± 9.24		
	41–50	137	19.9%	44.69 ± 9.65		
	≥ 51	119	26.1%	45.20 ± 9.18		
Marital status	Single	249	36.2%	44.59 ± 9.18	1.209	0.306
	Married	413	60.1%	44.78 ± 8.85		
	Divorced	18	2.6%	41.04 ± 7.43		
	Others	7	1.1%	47.32 ± 12.34		
Education	Junior high school or below	295	42.9%	46.36 ± 9.22	7.701	*P*<0.001
	Senior high school or technical secondary school	196	28.5%	43.95 ± 8.63		
	Junior college or College	179	26.1%	42.49 ± 8.40		
	Postgraduate or above	17	2.5%	45.51 ± 9.57		

Note: The SAS is the self-Rating Anxiety Scale score

### The relationship between insomnia, psychological capital, self-esteem and anxiety

The results of the correlation analysis for the four variables are shown in Table 2. Insomnia in COVID-19 patients was negatively correlated with psychological capital (r = -0.19, *P* < 0.001) and self-esteem (r = -0.22, *P* < 0.001). Insomnia was positively correlated with anxiety (r = 0.41, *P* < 0.001). Psychological capital was positively correlated with self-esteem (r = 0.53, *P* < 0.001). Anxiety was negatively correlated with psychological capital (r = -0.36, *P* < 0.001) and self-esteem (r = -0.49, *P* < 0.001).

**Table 2:** Correlation analysis of insomnia, psychological capital, self-esteem and anxiety

Variables	Mean ± standard deviation	1	2	3	4	5	6	7	8
1 Insomnia	6.044±4.663	1							
2 Self-effificacy	26.879±5.639	-0.179***	1						
3 Hope	26.328±5.447	-0.165***	0.773***	1					
4 Resilience	25.983±4.586	-0.158***	0.729***	0.778***	1				
5 Optimism	25.201±4.058	-0.146***	0.512***	0.551***	0.604***	1			
6 Psychological capital	104.390±17.127	-0.188***	0.892***	0.912***	0.898***	0.743***	1		
7 Self-esteem	28.993±3.984	-0.218***	0.483***	0.448***	0.488***	0.408***	0.529***	1	
8 Anxiety	44.642±8.981	0.410***	-0.310***	-0.248***	-0.355***	-0.357***	-0.361***	-0.493***	1

Note: *** *P*<0.001.

### Mediating effect test

As shown in Table 3, the SPSS macro program Process, compiled by Hayes, was used to perform the mediation effect analysis. Under the condition of controlling gender, age, marital status and education background, the mediating effect of psychological capital and self-esteem on the relationship between insomnia and anxiety in COVID-19 patients was analyzed. Regression analysis showed that insomnia had a direct negative predictive effect on psychological capital (β = -0.70, *P* < 0.001) and self-esteem (β = -0.13, *P* < 0.001). Psychological capital had a direct positive predictive effect on self-esteem (β = 0.12, *P* < 0.001). Insomnia had a direct positive predictive effect on anxiety (β = 0.61, *P* < 0.001). Both psychological capital and self-esteem had significant negative predictive effects on anxiety (β = -0.06, *P* < 0.05; β = -0.72, *P* < 0.001).

**Table 3:** The relationship between insomnia and anxiety:the chain mediating effect of psychological capital and self-esteem

Regression equation		Overall fit index			Significance of regression coefficient			
Outcome Variable	Predictor variable	R	R^2^	F	β	SE	t	*P*
Psychological capital	insomnia	0.209	0.044	6.238***	-0.699	0.140	-5.006	*P* < 0.001
	Gender				-1.176	1.338	-0.879	0.380
	Age (years)				0.092	0.064	1.446	0.149
	Marital status				0.677	1.386	0.488	0.625
	Education				1.192	0.754	1.586	0.113
Self-esteem	insomnia	0.596	0.355	62.416***	-0.129	0.028	-4.757	*P* < 0.001
	psychological capital				0.116	0.007	15.849	*P* < 0.001
	Gender				0.910	0.256	3.557	*P* < 0.001
	Age (years)				-0.019	0.012	-1.573	0.116
	Marital status				0.602	0.265	2.271	0.024
	Education				0.959	0.145	6.636	*P* < 0.001
Anxiety	insomnia	0.600	0.359	54.416***	0.611	0.062	9.833	*P* < 0.001
	psychological capital				-0.060	0.019	-3.127	0.002
	Self-esteem				-0.722	0.086	-8.957	*P* < 0.001
	Gender				0.621	0.581	1.069	0.286
	Age (years)				0.024	0.027	0.884	0.377
	Marital status				-1.110	0.598	-1.856	0.064
	Education				-0.986	0.336	-2.942	0.003

Note: *** *P* < 0.001.

**Fig. 1:**
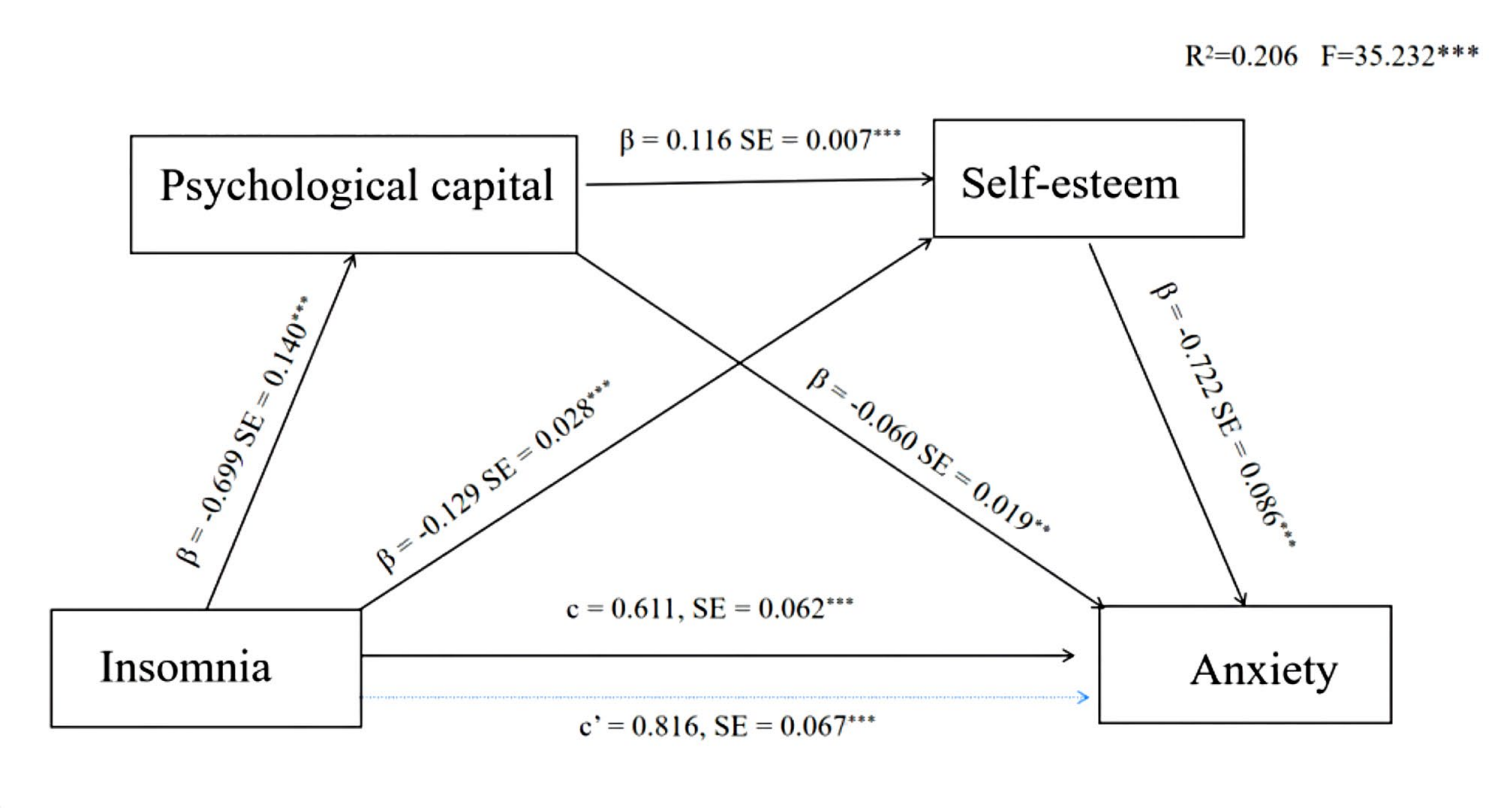
Serial-multiple mediation of psychological capital and self-esteem in the relationship between insomnia and anxiety with non-standardized beta values and standard error Note: ***P < 0.001, ** *P* less than 0.05

As shown in Table 4, the Mediation model was tested according to the method proposed by Hayes (9) The Bootstrap method was used to repeatedly extract 10,000 times to calculate 95% confidence intervals to further test the mediating effect. The results showed that the mediating effect of psychological capital and self-esteem was significant, and the mediating effect value was 0.21. Specifically, the mediation effect was generated through three mediation chains. First, the indirect effect 1 consisting of insomnia - psychological capital - anxiety was 0.04, and the Bootstrap 95% confidence interval did not include 0, indicating the significant mediating effect of psychological capital. Second, the indirect effect 2 consisting of insomnia-self-esteem-anxiety had a value of 0.10 with a Bootstrap 95% confidence interval that did not include 0, indicating that self-esteem had a significant mediating effect. Third, the indirect effect 3 consisting of insomnia-psychological capital-self-esteem-anxiety had a value of 0.06 and the Bootstrap 95% confidence interval did not contain a zero, suggesting that psychological capital and self-esteem had a significant chain mediating effect between insomnia and anxiety. The specific pathways of insomnia in COVID-19 patients acting on anxiety are illustrated in Fig. 1.

**Table 4:** Comparison of indirect effects of insomnia on anxiety mediated by psychological capital and self-esteem

	Product of Coefficients		Bootstrapping 95% BC Confidence Interval(CI)		Proportion of indirect effect
	Point Estimate	Boot SE	BootLL CI	BootUL CI	
Effect					
Total indirect effect of X on Y	0.205	0.035	0.138	0.276	25.25%
Indirect effect 1: X→M1→Y	0.042	0.018	0.013	0.080	5.16%
Indirect effect 2: X→M2→Y	0.100	0.023	0.056	0.145	12.26%
Indirect effect 3: X→M1→M2→Y	0.063	0.017	0.031	0.100	7.72%
Contrasts					
Model 1 versus Model 2	-0.058	0.031	-0.117	0.005	
Model 1 versus Model 3	-0.021	0.021	-0.066	0.018	
Model 2 versus Model 3	0.037	0.027	-0.018	0.088	

Note: N = 687. Number of bootstrap samples for bias corrected bootstrap confidence intervals: 10,000. Level of confidence for all confidence intervals: 95%

X = insomnia, M1 = psychological capital, M2 = self-esteem, Y = anxiety. Model 1 = insomnia - psychological capital - anxiety; Model 2 = insomnia - self-esteem -  anxiety; Model 3 = insomnia - psychological capital - self-esteem - anxiety. LL = lower level; UL = upper level

## Discussion

This study found that insomnia had a significant positive predictive effect on anxiety, and that psychological capital and self-esteem had a chain mediating effect on the relationship between insomnia and anxiety. The higher the level of insomnia in COVID-19 patients, the lower their psychological capital level, which in turn led to lower self-esteem, which further led to higher anxiety status.

### Direct effect of insomnia on anxiety

This study found that insomnia positively predicted anxiety, which verified the direct path of insomnia to anxiety, indicating that the higher the degree of insomnia, the higher the level of anxiety, similar to the results of a meta-analysis of six observational studies by Elisabeth, which showed that insomnia was a major predictor of anxiety attacks (odds ratio 3.2) [13].

Insomnia and its accompanying symptoms are associated with certain neurotransmitters, such as the neurotransmitter 5-HT, which is involved in the function of the sleep-wake system. 5-HT is secreted by the hypothalamus, which regulates the body’s emotional physiological responses and the functioning of the sleep and wake systems [24]. Serum 5-HT level can reflect the central activity state, and reduced 5-HT secretion will cause insomnia, anxiety and depression [24, 58]. Similarly, studies have shown that low-frequency acupoint electrical stimulation has a certain effect on insomnia patients after stroke, and it also regulates sleep by affecting the plasma 5-HT level [21]. It is suggested that we can adjust the immune function of the body by dredging the meridians and collaterals, and then regulate the endocrine, to improve the quality of sleep and promote the synthesis and release of monoamine neurotransmitters or reduce their degradation, and increase the secretion of 5-HT in the hypothalamus, so as to achieve the purpose of improving sleep and relieving anxiety. Simultaneously, for patients with COVID-19, in addition to the stress and anxiety caused by the disease itself, the anxiety may be aggravated by concentrated isolation treatment, crowded people, unfamiliar environment, depressed environment in the ward, economic burden, social isolation, family members and relatives suffering from COVID-19 or getting worse, or death due to COVID-19 [13]. Therefore, it is necessary to pay extra attention to the psychology of COVID-19 patients and try to meet their reasonable needs.

Anxiety is the most common mental health problem in the world. Epidemiological studies showed that about 50% of patients with anxiety disorders were affected by sleep disorders, especially insomnia, and lack of sleep could trigger or further aggravate anxiety disorders [13]. Therefore, it is necessary to recognize the negative influence of anxiety caused by insomnia, to find the comorbidities that manifest as insomnia, to assess and manage in a timely manner, to detect early, and to intervene early in order to reduce the risk of future development and promote better sleep quality and duration. In order to avoid a series of adverse effects, especially anxiety [7].

### Mediating mechanism of psychological capital

This study found that insomnia negatively predicted psychological capital, and psychological capital negatively predicted anxiety. Psychological capital had a mediating effect between insomnia and anxiety (accounting for 5.16%), which verified the mediating path of psychological capital between insomnia and anxiety. It indicated that insomnia significantly negatively predicted psychological capital, and psychological capital significantly negatively predicted anxiety level.

Psychological capital is a positive mental state of individuals in the process of growth [26]. Insomnia is a subjective experience in which insomniacs are not satisfied with sleep time and/or sleep quality. When insomnia, a common sleep disorder, occurs for a long time, patients will pay selective attention to sleep-related concerns and threats, and have false perceptions about sleep, such as excessive sleep demands, excessive negative thoughts about the impact of sleep on daytime function, and so on. Not only does it reduce quality of life and affect social functioning, it causes a wide range of physical and mental illnesses and reduces individual self-efficacy and hope. In daily life, self-efficacy decreases, that is, when completing a specific task in a specific situation, the degree of trust in their ability to stimulate their own motivation, use cognitive resources and execute action plans decreases, and individuals with low self-efficacy are more likely to have tension and anxiety [41]. Studies have shown that better psychological resilience, perceived greater social support and a positive coping style are protective factors for depression and anxiety symptoms. Living alone, major physical diseases and negative coping were risk factors for depression, anxiety symptoms and insomnia [20]. In the correction of anxiety caused by insomnia, positive guidance and other psychological interventions could relieve patients’ negative emotions, and improve patients’ psychological capital such as self-efficacy and hope in the face of the disease, to avoid the generation of irritability, dissatisfaction, anxiety and other emotions caused by negative suggestions and poor self-evaluation [37]. Studies have shown that psychological resilience could regulate the influence of insomnia on anxiety, which indicates that in the treatment process of anxiety caused by insomnia, psychological capital such as psychological resilience can be improved to regulate bad emotions and thus improve the anxiety of patients [52]. Individuals are advised to consciously develop psychological capital to face work and life more positively, optimistically and resolutely, and to cope effectively with various pressures to improve mental health.

### Mediating mechanism of self-esteem

Self-esteem had a mediating effect between insomnia and anxiety (accounting for 12.26%), which verified the mediating pathway of self-esteem between insomnia and anxiety. It indicated that insomnia level significantly negatively predicted self-esteem, and self-esteem significantly negatively predicted anxiety level, which is almost the same as the research of Woods and Scott [51].

Self-esteem is an individual’s perception of his or her emotions. It is an emotional experience and refers to the difference between the real self and the ideal self that the individual perceives. The self-esteem level determines whether an individual has a positive opinion of himself or herself. Individuals with strong self-esteem are able to show self-acceptance, and like themselves and acknowledge their own value, while individuals with low self-esteem perceive themselves as incompetent, annoying, and deny their worth [1]. There is a strong and significant correlation between self-esteem and anxiety, that is, the higher the level of self-esteem, the lower the level of anxiety, and vice versa [11]. Stigma is the phenomenon of psychological and social maladjustment caused by a person suffering from a disease, accompanied by negative self-perceptions. As a common psychological problem, Stigma widely exists in chronic diseases [3]. And because of the complex course of treatment for the disease itself, as well as the prolonged treatment time and accompanying complications, COVID-19 insomniacs not only cause great psychological stress to patients and families, but also great suffering. If patients integrate and internalize these illness experiences and negative emotions, a sense of stigma will be formed in their psychology, which will lead to a decline in self-esteem [34], and further increase negative emotions such as inferiority complex, anxiety and depression. It indicates that self-esteem is significantly correlated with mental health and physical health [7].

Cognitive behavioral intervention is a psychological and behavioral treatment method, which can achieve the purpose of eliminating patients’ adverse cognition, emotion and behavior through changing the thinking and behavior and correcting pathological views of patients, to improve self-esteem, so as to significantly improve anxiety and depression, to promote patients’ physical and mental health [55]. This therapy was effective regardless of short-term or long-term use and had a long-lasting effect, so it is recommended as the first-line treatment for insomnia in domestic and foreign guidelines [36]. It is therefore necessary to encourage the patient to brave sleeplessness and a calm acceptance of reality in order to relax and maintain a steady mood; Empower patient self-recognition, attention and appreciation in clinical practice; Find them self-worth and give them some social support to improve their psychological resilience and reduce the occurrence of anxiety.

### Chain mediating effect mechanism of psychological capital and self-esteem

This study found that the chain mediating effect of psychological capital and self-esteem on the relationship between insomnia and anxiety was 0.06 (7.72%), that is, insomnia could have a significant influence on anxiety through the chain mediating effect of psychological capital and self-esteem, which verified the chain mediating path of psychological capital and self-esteem. This suggests that psychological capital not only acts as a single mediator between insomnia and anxiety, but also further influences anxiety by affecting self-esteem levels.

It can be seen from the results that self-esteem was positively correlated with all dimensions of psychological capital (self-efficacy, hope, resilience and optimism), which is basically consistent with existing conclusions [32]. When encountering COVID − 19, due to the lack of effective and timely supporting resources, they had to consume their own positive psychological resources, negative evaluating their own ability and value, reducing self-confidence, producing mental pessimistic disappointment, so that psychological capital of patients with insomnia dropped, as well as mental resilience and self-efficacy [50]. However, self-esteem level is related to individual resilience, self-efficacy and internal strength, reflecting their ability to effectively cope with adversity [27, 50]. A large number of applied studies have shown that mindfulness-based cognitive therapy has therapeutic effects on a variety of mental disorders, which can significantly improve the self-esteem level, self-harmony level and reduce anxiety level of insomnia patients [46]. It is a psychotherapy that reduces or eliminates adverse emotions and behaviors by modifying patients’ cognitive patterns, which can effectively correct patients’ irrational cognition, improve their psychological adjustment ability, and thus relieve anxiety symptoms [56]. According to the above theory, it is necessary to pay attention to temporal changes in the physical and mental conditions of COVD-19 patients during clinical work. At the same time, the psychological capital status of patients needs to be evaluated to improve self-esteem levels to reduce anxiety in COVID-19 patients with insomnia.

The COVID-19 epidemic has led to the decline of the global economy, information lag in some regions, the body interference by diseases, and some patients are separated from their families due to isolation, which may be more likely to have psychological anxiety. Therefore, we should pay more attention to the psychological problems of COVID-19 patients as a vulnerable group.

There are some limitations to this study as well. First, the cross-sectional study design does not confirm causality between variables, which still needs to be explored in longitudinal studies. The study was conducted in only one hospital with centralized management of COVID-19 patients. Management systems and shift patterns at different hospitals may have an impact on the variables. Investigations should be conducted in different regions or other hospitals to confirm the results of this study in the future. Third, among numerous mental health outcomes, we only assessed insomnia and anxiety in COVID-19 patients. However, we believe that the relationships between variables confirmed by the results of this study are also present in depression and even in other occupations.

## Conclusion

The present study is the first to explore the relationship between insomnia and anxiety in COVID-19 patients in China using a serial multiple mediation model. The results of the present study suggest that a reduction in insomnia is sequentially associated with an increase in psychological capital, followed by an increase in self-esteem, which in turn is associated with a reduction in anxiety. Therefore, focusing on improving the psychological capital and self-esteem of patients can help alleviate the anxiety caused by insomnia in COVID-19 patients.

## Data Availability

De-identified textual data can be made available upon reasonable request to the corresponding author.

## Abbreviations

SES: Self-esteem scale
COR: Conservation of Resources theory
SAS: Self-Rating Anxiety Scale
AIS: Athens Insomnia Scale
